# Exploitation of Endophytic Bacteria to Enhance the Phytoremediation Potential of the Wetland Helophyte *Juncus acutus*

**DOI:** 10.3389/fmicb.2016.01016

**Published:** 2016-07-04

**Authors:** Evdokia Syranidou, Stavros Christofilopoulos, Georgia Gkavrou, Sofie Thijs, Nele Weyens, Jaco Vangronsveld, Nicolas Kalogerakis

**Affiliations:** ^1^School of Environmental Engineering, Technical University of CreteChania, Greece; ^2^Centre for Environmental Sciences, Hasselt UniversityDiepenbeek, Belgium

**Keywords:** wetland plant, *J. acutus*, endophytic bacteria, metals, emerging organic contaminants

## Abstract

This study investigated the potential of indigenous endophytic bacteria to improve the efficiency of the wetland helophyte *Juncus acutus* to deal with a mixed pollution consisting of emerging organic contaminants (EOCs) and metals. The beneficial effect of bioaugmentation with selected endophytic bacteria was more prominent in case of high contamination: most of the inoculated plants (especially those inoculated with the mixed culture) removed higher percentages of organics and metals from the liquid phase in shorter times compared to the non-inoculated plants without exhibiting significant oxidative stress. When exposed to the lower concentrations, the tailored mixed culture enhanced the performance of the plants to decrease the organics and metals from the water. The composition of the root endophytic community changed in response to increased levels of contaminants while the inoculated bacteria did not modify the community structure. Our results indicate that the synergistic relationships between endophytes and the macrophyte enhance plants’ performance and may be exploited in constructed wetlands treating water with mixed contaminations. Taking into account that the concentrations of EOCs used in this study are much higher than the average contents of typical wastewaters, we can conclude that the macrophyte *J. acutus* with the aid of a mixed culture of tailored endophytic bacteria represents a suitable environmentally friendly alternative for treating pharmaceuticals and metals.

## Introduction

Emerging organic contaminants (EOCs) consist of a wide range of synthesized and natural compounds, such as pharmaceuticals, personal care products, surfactants, and industrial chemicals (Luo et al., 2014). Conventional wastewater treatment plants are the main point sources of their release into the environment since these systems have not been designed to treat these kinds of chemical products (Jiang et al., 2013). EOCs are discharged into surface waters, wherein they can cause toxicity already at low concentrations (LCs; Matamoros et al., 2016) and change the microbial ecosystems (Baquero et al., 2008). Huang et al. (2012) identified multiple antibiotic resistant bacteria in secondary eﬄuents of a municipal wastewater treatment plant in China, posing the risk of dispersion of antibiotic genes. In another study, Hu et al. (2010) demonstrated that several antibiotics are spread in the environment and food-chains through water or even absorption in vegetables that are fertilized by manure.

Constructed wetlands (CWs) are reliable green engineered systems that are employed for treating different kinds of eﬄuents such as domestic, industrial or agro-industrial wastewater, and acid mine drainage (Wu et al., 2014). These systems take advantage of the ability of plants together with their associated microorganisms to remove organic xenobiotics and metals from the water. At the same time with the biological processes, complex physical and chemical processes contribute to the degradation/detoxification/elimination of contaminants (Zhang et al., 2014). Several studies have revealed the effective role of aquatic plant- and microbe-based systems in the removal of emerging contaminants (Ávila et al., 2013; Verlicchi and Zambello, 2014; Vymazal and Březinová, 2015).

Selection of the appropriate macrophyte(s) in CWs is of high importance because plants play a significant role in pollutant removal by direct mechanisms or by enhancing the degradation activity of the microorganisms in the rhizosphere (Li et al., 2014). Species like *Typha* spp., *Cyperus alternifolius*, and *Phragmites australis* were shown to efficiently remediate EOCs contaminated water (Dordio et al., 2011; Liu et al., 2013; Yan et al., 2016). However, these EOCs may cause toxicity to the plants thus decreasing their performance. A potential strategy to overcome this problem is the exploitation of endophytic bacteria. These microorganisms reside in the internal plant tissues without negatively affecting the host plant and often are involved in mutualistic relationships. Endophytes are known to possess PGP traits and degradation genes that assist their host plant to cope with various environmental stresses. Once they are inside, they can contribute *in planta* to the detoxification of organic contaminants by degrading them and/or enhance the metal translocation providing a potential toward sustainable treatment of mixed contaminations (Babu et al., 2013; Ho et al., 2013; Visioli et al., 2015). There exist a limited number of studies that highlight the use of endophytic bacteria in terms of enhancing the performance of wetlands treating sewage eﬄuent (Ijaz et al., 2015) or textile eﬄuent (Shehzadi et al., 2014).

*Juncus acutus* is a helophyte widely used in CWs in Europe and North America (Vymazal, 2013). The aim of this study is to investigate the potential of indigenous endophytic bacteria to improve the efficiency of the wetland plant *J. acutus* in dealing with EOCs, one endocrine disruptor (bisphenol A, BPA) and two antibiotics [ciprofloxacin (CIP), sulfamethoxazole (SMX)] and metals (zinc, nickel, cadmium). The oxidative stress that is induced in plants was assessed measuring the activities of enzymes involved in cellular defense against oxidative stress [syringaldazine peroxidases (SPOD), glutathione reductases (GR), guaiacol peroxidases (GPOD), and superoxide dismutases (SOD)], among the different treatments in order to evaluate the impact of the contaminants on the plant. The potential consequences of inoculation on the root microbial community were also determined.

## Materials and Methods

### Selection of Most Promising Bacterial Strains

#### Bacterial Strains

The bacterial strains used in this study were isolated from roots and leaves of the wetland plant *J. acutus* growing on a BPA-contaminated pilot (Syranidou et al., 2016). The strains (shown in **Table 1**) were selected based on their *in vitro* plant growth promoting (PGP) abilities [phosphate solubilization, production of indoleacetic acid (IAA), ACC-deaminase (ACC), organic acids, and siderophores], tolerance to metals (Zn, Ni, Cd) and emerging contaminants (BPA, CIP, SMX), and potential for degradation of these contaminants. All the tests were described and performed previously (Syranidou et al., 2016).

**Table 1:** Isolated endophytic bacteria based on traits and corresponding treatments (P Solub, phosphate solubilizers; IAA, indoleacetic acid producers; ACC, ACC deaminase producers; OA, organic acid producers; Sid, siderophores producers; Zn, zinc; Ni, nickel; Cd, cadmium; BPA, bisphenol A; CIP, ciprofloxacin; SMX, sulfamethoxazole; TET, tetracycline; E, erythromycin; x, positive in the tested characteristic; –, not possessing the tested characteristic) from Syranidou et al. (2016).

ID	Description of treatment (inoculations)	Isolated from	Promoting traits					Metal tolerance			EOCs tolerance					EOCs potential degradation				
			P solub	ACC	IAA	OA	Sid	4 mM Zn	1 mM Ni	1 mM Cd	BPA	CIP	SMX	TET	E	BPA	CIP	SMX	TET	E
NIN	Non-inoculated	–	–	–	–	–	–	–	–	–	–	–	–	–	–	–				
IN1	*Microbacterium* sp. U50/*Microbacterium* sp. R31	Leaves/roots	x/x	x/–	x/x	x/x	–/x	x/x	x/x	x/x	x/x	x/x	x/x	–/–	–/–	x/x	–/–	–/–	–/–	–/–
IN2	*Herbaspirillum* sp. L32	Leaves	–	x	x	–	x	x	x	x	x	–	–	–	–	–	–	–	–	–
IN3	*Yonghaparkia* sp. R2b	Roots	–	x	x	x	x	x	x	–	–	–	–	–	–	–	–	–	–	–
IN4	*Sphingomonas* sp. U33 (also denoted as Bl)	Leaves	x	x	x	–	–	x	x	x	x	x	x	–	–	x	–	–	–	–
IN5	*Bacillus* sp. R12 (also denoted as B2)	Roots	x	–	–	–	x	x	x	–	x	–	–	–	–	x	–	–	–	–
IN6	*Ochrobactrum* sp. R24 (also denoted as B3)	Roots	–	x	x	–	–	x	x	x	x	–	x	–	–	x	–	x	–	–
IN7	*Acidovorax* sp. U3	Leaves	–	x	x	–	–	x	x	x	x	–	x	–	–	x	–	–	–	–
IN8	*Ralstonia* sp. U42/*Ralstonia* sp. R52	Leaves/roots	x/x	x/–	x/x	–/x	x/x	x/x	x/x	x/x	x/x	–/–	–/x	–/–	–/–	x/–	–/–	–/–	–/–	–/–
IN9	*Pseudomonas* sp. R15	Roots	x	–	–	–	x	x	x	–	x	–	x	–	–	–	–	–	–	–

#### *In vivo* Plant Growth Promotion

In a 1-month greenhouse experiment, the 11 most promising strains, based on their *in vitro* traits, were inoculated (concentration: 10^8^ cfu mL^-1^) to glass beakers with *J. acutus* plants (*n* = 10) and vermiculate as substrate. Young *J. acutus* plants were collected from Souda Bay (Chania, Greece). The control treatment consisted of plants without inoculation. The system was irrigated with 30 mL tap water every week and at the end of the experiment the root and shoot fresh and dry weights, and the root length were determined. Based on the results, the three strains with the highest *in vivo* plant growth promotion were selected for an inoculation experiment upon exposure to mixed contamination.

### Effect of Inoculation in the Presence of Mixed Contamination

#### Experimental Set-Up

*Juncus acutus* plants were collected from Souda Bay at Chania (Greece) and were acclimatized for 2 months in a greenhouse. Subsequently, plants (∼20 g fresh weight) were transferred to glass beakers with small-size gravel (0.2–0.5 cm) as substrate and the system was irrigated with 50 mL tap water every week. After 2 weeks, the three best growth promoting endophytic strains with different degradation capacities (B1—*Sphingomonas* sp. U33, B2—*Bacillus* sp. R12, B3—*Ochrobactrum* sp. R24) were inoculated (concentration: 10^8^ cfu mL^-1^) separately and as a consortium to the beakers (*n* = 10 for every treatment). The endophytic strains were cultured in 869 medium until the late log-phase, washed three times in 10 mM MgSO_4_ and resuspended in sterile water to reach an inoculum concentration of approximately 10^9^ cfu mL^-1^. One week later, two different concentrations of metals (Zn, Ni, Cd), BPA and two antibiotics: CIP and SMX were added. More specifically, 50 μg L^-1^ CIP, 250 μg L^-1^ SMX, 5 mg L^-1^ BPA, 200 mg L^-1^ Zn, 20 mg L^-1^ Ni, and 1 mg L^-1^ Cd were added to the LC treatments and 100 μg L^-1^ CIP, 500 μg L^-1^ SMX, 10 mg L^-1^ BPA, 400 mg L^-1^ Zn, 40 mg L^-1^ Ni, and 2 mg L^-1^ Cd were added to the high concentration (HC) treatments. A two-factorial study design was followed with factor 1 contaminant concentration (two levels, LC and HC), and factor 2 bioaugmentation treatments (five levels, no inoculation, strain 1, strain 2, strain 3, consortium). In total, there were five different treatments (one non-inoculated control and four bioaugmented treatments) concerning the inoculation effect and two different concentrations of the mixture of contaminants (one LC and one HC) concerning the contamination effect (**Table 2**). The experiment lasted for 21 days and was irrigated with 50 mL tap water every week.

**Table 2:** Experimental design—treatments examined.

ID	Description of contaminants	Controls (not-inoculated)	Leaf-endophyte	Root-endophyte	Root-endophyte	Mixed culture
		C	B1	B2	B3	MIX
NC	No contamination	C_NC				
LC	50 μg L^-1^ ciprofloxacin (CIP), 250 μg L^-1^ sulfamethoxazole (SMX), 5 mg L^-1^ bisphenol A (BPA), 20 mg L^-1^ Ni, 1 mg L^-1^ Cd, 200 mg L^-1^ Zn	C_LC	B1_LC	B2_LC	B3_LC	MIX_LC
HC	100 μg L^-1^ CIP, 500 μg L^-1^ SMX, 10 mg L^-1^ BPA, 40 mg L^-1^ Ni, 2 mg L^-1^ Cd, 400 mg L^-1^ Zn	C_HC	B1_HC	B2_HC	B3_HC	MIX_HC

#### Sampling

Water samples were taken at days 0, 14, and 21 after addition of the contaminants and were analyzed for their concentrations of metals and organic contaminants. The soluble metals were determined by inductively coupled plasma mass spectrometry (7500cx coupled to Autosampler Series 3000, both from Agilent Technologies). BPA, CIP, and SMX concentrations were measured by high-performance liquid chromatography (HPLC) (Shimadzu Corp., Kyoto, Japan), equipped with LC-10 ADVP solvent delivery module, SPD-M10 AVP Diode Array Detector, RF-10AXL Fluorescence Detector, and SIL-10 ADVP autosampler. Separation of BPA was accomplished on a Nucleosil 100–5 C-18 column and separation of CIP and SMX was performed on an Alltech Prevail^TM^ Organic Acid 5u as previously described by Christofilopoulos et al. (2016).

At the end of the experiment, the plants were harvested and washed first with water and then with distilled water. The fresh weights of roots and leaves were determined, plant parts were cut in small pieces and 0.4 g of each plant compartment was sampled for enzymatic analysis while 0.3 g of roots was taken for DNA extraction. The plant samples for further analysis were immediately snap-frozen in liquid nitrogen and stored at -80°C; the remaining material was dried at 45°C and weighed.

#### Enzyme Assays

The activities of the antioxidative enzymes were assessed in order to estimate the oxidative stress induced in the plants (*n* = 10). Fresh leaves and roots were macerated in liquid nitrogen and then homogenized in 0.1 M Tris–HCl buffer (pH 7.8) containing 1 mM 1,4-dithiothreitol and 1 mM ethylenediaminetetraacetic acid. The homogenate was centrifuged at 13,500 rpm and 4°C for 10 min and the supernatant was used for the enzyme analysis. The activity of GR that catalyze the reduction of oxidized glutathione (GSSG) to reduced glutathione (GSH) by oxidizing NADPH was measured at 340 nm and the activity of GPOD was estimated at 436 nm by the appearance of tetra-guaiacol (Bergmeyer et al., 1974). The SPOD involved in H_2_O_2_ scavenging, were determined by the oxidation of syringaldazine at 530 nm (Imberty et al., 1985). Superoxide dismutases (SOD) deal with superoxide anions and were determined by following the cytochrome *c*-inhibition at 550 nm (McCord and Fridovich, 1969). All enzyme activities were expressed as units per gram fresh weight.

#### DNA Extraction and Endophytic Bacterial Community Profile

The fresh plant roots were immersed in 70% ethanol solution for 30 s and subsequently in 2% NaClO solution supplemented with one droplet Tween 80 per 100 mL solution for 10 min. The surface-sterilized plant parts were washed three times with distilled water for 1 min and 100 μL of the last rinsing solution were streaked on 869 plates (Mergeay et al., 1985) and incubated for 7 days at 30°C. The absence of colonies on the plates confirmed the successful disinfection. Next, the samples were macerated with liquid nitrogen and total DNA was extracted using the Invisorb^®^ Spin Plant Mini Kit (STRATEC Molecular GmbH, Berlin, Germany).

Automated ribosomal intergenic spacer analysis (ARISA) PCR was performed in order to estimate the root bacterial diversity and community structure among the different treatments. The primers ITSF (5′-GTCGTAACAAGG TAGCCGTA-3′) and ITSReub (5′-GCCAAGGCATCCACC-3′) were used for the amplification of the intragenic transcribed region ITS1 in the rRNA operon plus ca. 282 bases of the 16S and 23S rRNA (Cardinale et al., 2004). A mixture of 0.2 mM of each of the four deoxynucleoside triphosphates, 2 mM MgCl_2_, 0.2 μM each of the forward and reverse primers and 1 U of High Fidelity Platinum Taq DNA polymerase per 25 μL was used to perform the PCR. The cycling conditions of the PCR were: one denaturation cycle at 94°C for 3 min, followed by 30 cycles at 94°C for 45 s, 56°C for 45 s, 72°C for 2 min, and a final extension at 72°C for 7 min.

The gel–dye mix, marker, PCR products, and ladder were loaded to the DNA chip according to the manufacture’s protocol (Agilent DNA 1000 Assay Protocol), next the chip was inserted to the 2100 Bioanalyzer (Agilent Technologies, Diegem, Belgium) and the chip run was executed.

### Data Analysis

The statistical analysis was performed with the automatic R (R Development Core Team, 2009); ANOVA and non-parametric tests were applied to data that follow and did not follow normal distribution, respectively. Concerning the contaminants, a two-way ANOVA was performed and the results indicated an interaction effect between the level of contamination and inoculation, so the main effects of the independent variable were investigated separately. Next, analysis of ARISA fragments was performed with Bioanalyzer software. Only peaks with sizes between 100 and 1500 bp and a minimum peak height of 150 fluorescence units were considered for analysis. The binning of ARISA fragments was performed according to Ramette (2009). Briefly, the automatic R binning script was applied to replicates of the same treatment in order to find the window size (WS) and the shift value and a WS of 1 bp was selected for the operational taxonomic unit (OTU) binning algorithm for ARISA profiles of endophytic bacteria. The analysis of the OTU table was performed by Primer 6 software. A multidimensional scaling (MDS) plot was used to describe the root community structure while the degree of similarity was explored with the permutation-based hypothesis statistical test analysis of similarity (ANOSIM).

## Results

### Selection of Most Promising Bacterial Strains

**FIGURE 1:**
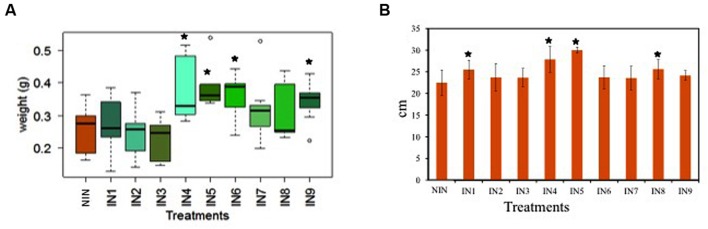
**Box-plot with the dry weight of the plantlets (A), the root length of the plantlets (B); NIN, non-inoculated; IN1, inoculated with *Microbacterium* sp. U50/*Microbacterium* sp. R31; IN2, inoculated with *Herbaspirillum* sp. L32; IN3, inoculated with *Yonghaparkia* sp. R2b; IN4, inoculated with *Sphingomonas* sp. U33; IN5, inoculated with *Bacillus* sp. R12; IN6, inoculated with *Ochrobactrum* sp. R24; IN7, inoculated with *Acidovorax* sp. U3; IN8, inoculated with *Ralstonia* sp. U42/*Ralstonia* sp. R52; IN9, inoculated with *Pseudomonas* sp. R15 [error bars are ± standard error (*n* = 10), data with asterisk are significantly different (*p* < 0.05) compared to the non-inoculated plants]**.

The endophytic strains with the best results for *in vitro* PGP traits (**Table 1**) were tested *in vivo* for their capability to enhance the plant biomass production. Eleven strains were inoculated in pots with young *J. acutus* plants (11.3 cm leaf height, 0.44 g fresh biomass) in a 1-month greenhouse experiment. The effect of inoculation on plant weight varied among the treatments (**Figure 1**). Overall, some endophytic strains increased the plant weight in comparison to the non-inoculated controls. More specifically, the dry weight of the plants inoculated with *Sphingomonas* sp. U33 (IN4), *Bacillus* sp. R12 (IN5), *Ochrobactrum* sp. R24 (IN6), and *Pseudomonas* sp. R15 (IN9) was statistically (*p* < 0.05) higher in comparison to the dry weight of the non-inoculated plants (**Figure 1**). The root lengths of the inoculated plants with IN1, IN4, IN5, and IN8 were significantly higher (25.51, 27.87, 30.00, and 25.61 cm, respectively) in comparison to non-inoculated control plants (22.48 cm).

Considering the performance of endophytic strains in demonstrating PGP traits *in vivo*, together with their results concerning metal tolerance, BPA/CIP/SMX resistance and potential degradation (see **Table 1**), three strains (B1—*Sphingomonas* sp. U33, B2—*Bacillus* sp. R12, B3—*Ochrobactrum* sp. R24) were selected for further investigation of their effects on *J. acutus* under stress conditions. These three strains were tested separately and as a consortium.

### Removal of Organic Contaminants in Bioaugmented Microcosms

In order to investigate the removal efficiencies of the different microcosms, mixtures of organic contaminants were added to the pots.

**FIGURE 2:**
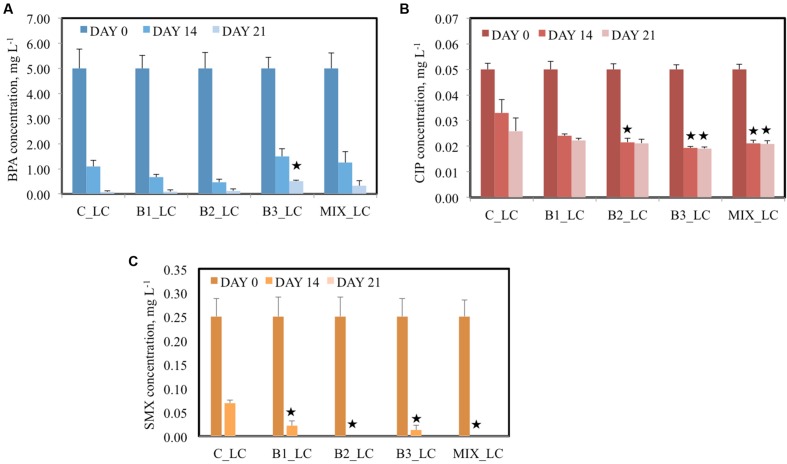
**Concentration of BPA **(A)**, CIP **(B)**, and SMX **(C)** of the different treatments after 0, 14, and 21 days when the low concentration (LC) of contaminants were added [data with asterisk are significantly different (*p* < 0.05) compared to the non-inoculated plants]**.

In LC treatments (LC of contaminants were added to the pots), 5 mg L^-1^ BPA was supplemented in each pot and after 14 days more than 70% BPA removal from the water was observed in all cases, independent of inoculation (**Figure 2A**). The highest percentage of removal was realized by the B2-inoculated plants (90.7%) while the B3-inoculated plants removed 70.1% BPA from the water. At the same sampling day, the non-inoculated plants realized a removal of 78.1% BPA from the surrounding water. After 21 days, BPA concentration was lower than 0.5 mg L^-1^ for the B3 and consortium-inoculated plants and less than 0.1 mg L^-1^ for the B1- and B2-inoculated and the non-inoculated plants. The non-inoculated plants removed the highest percentage (98.4%) of BPA in comparison with all the inoculated.

**FIGURE 3:**
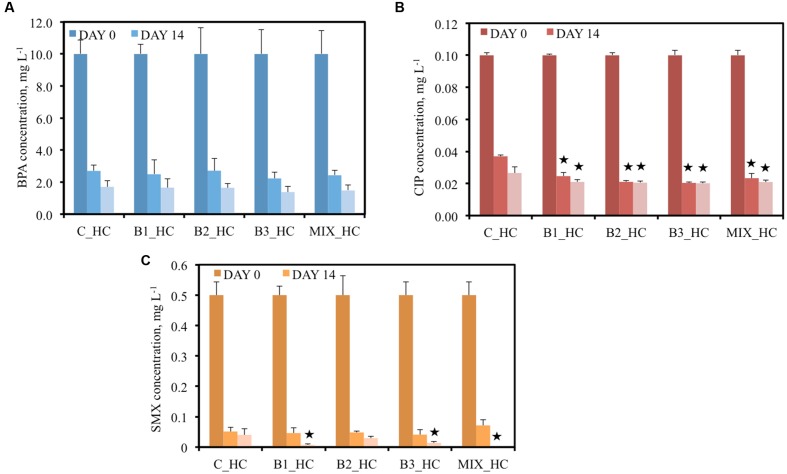
**Concentration of BPA **(A)**, CIP **(B)**, and SMX **(C)** of the different treatments after 0, 14, and 21 days when the high concentration (HC) of contaminants were added [data with asterisk are significantly different (*p* < 0.05) compared to the non-inoculated plants]**. The names of the bacteria are given at **Table 1**.

When 10 mg L^-1^ BPA was added to the pots, all plants showed a comparable capacity to remove BPA; thus, no statistically significant differences were observed among the treatments (**Figure 3A**). At day 14, BPA concentration was approximately 2.7 mg L^-1^ in the surrounding water of non-inoculated and B2-inoculated plants and less than 2.5 mg L^-1^ in the other inoculated plants. Similarly, 1.7 mg L^-1^ BPA remained in the water of plants with only their indigenous community, approximately 1.6 mg L^-1^ in the water of B1- and B2-inoculated pots, 1.48 mg L^-1^ in the water of plants inoculated with the consortium and 1.38 mg L^-1^ in the water of B3-inoculated plants after 21 days of inoculation. At that day, the lowest removal was observed for the non-inoculated plants (83%) while the highest was recorded for the B3-inoculated (86.2%) demonstrating that the differences in removal are minor.

The pots were also contaminated with two different concentrations of the antibiotic CIP, 0.05 mg L^-1^ in the LC and 0.1 mg L^-1^ in the HC treatments. After 14 days of incubation, significant decreases in antibiotic concentrations were detected in all water samples. Moreover, significant differences were observed between the samples of inoculated plants and the non-inoculated plants at the same time point (**Figure 2B**) in the LC treatments. For example, the CIP concentrations were decreased with 34 and 48.3% after 14 and 21 days, respectively, by the non-inoculated plants while all the inoculated plants realized decreases of more than 50% in CIP concentration after 14 days. In the HC treatments, the capacity of plants inoculated with endophytes to remove CIP was significantly higher than the non-inoculated plants since they removed more than 70% CIP in comparison with 63% after 14 days. At the end of the experiment, more than 79% removal of CIP was established by all inoculated plants and 73.5% by the non-inoculated plants (**Figure 3B**).

The ability of plants to remove SMX from the water seemed to be correlated to the inoculation effect in the LC treatment. At an initial concentration of 0.25 mg L^-1^, the non-inoculated plants removed 72.4% SMX from the surrounding water after 14 days while all the inoculated plants removed more than 90% (**Figure 2C**). Moreover, after 14 days SMX could not be detected anymore in samples inoculated with B2 and the consortium while after 21 days, SMX was below detection limit in all water samples. In the HC treatment the initial SMX concentration was the double (0.5 mg L^-1^); at day 14 all the plants showed similar removal efficiency (approximately 90%) but after 21 days the inoculated plants demonstrated a higher removal (**Figure 3C**), except in case of B2. It is worth noticing that at that sampling day, SMX was below detection limit in the water samples of consortium-inoculated plants.

### Removal of Metals by the Several Treatments

**FIGURE 4:**
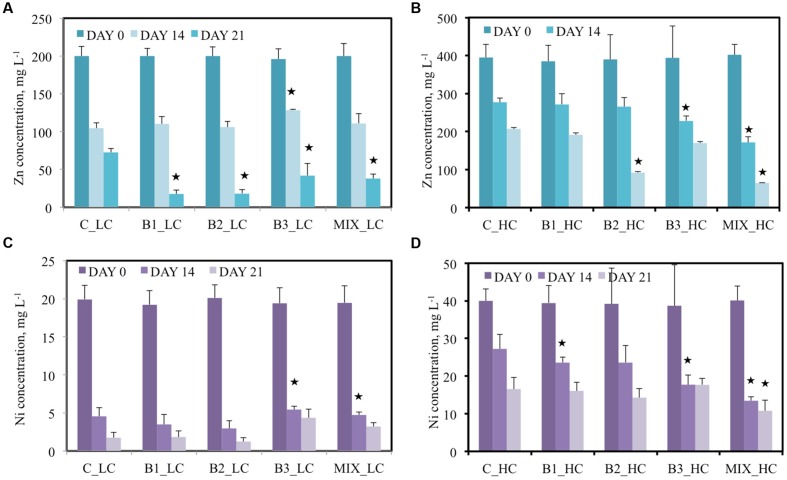
**Concentration of Zn **(A,B)** and Ni **(C,D)** of the different treatments after 0, 14, and 21 days when the low **(A,C)** and high **(B,D)** concentration of contaminants were added**. Data with asterisk are significantly different (*p* < 0.05 compared to the non-inoculated plants).

Besides the organic contaminants, the water in the pots was contaminated with zinc, nickel, and cadmium, like for the other contaminants at two different concentrations. In the LC treatments, 200 mg L^-1^ Zn was added and an approximately 45% decrease in concentration was recorded for most of the different treatments after 14 days (**Figure 4A**). The B3-inoculated plants showed the lowest efficiency to remove zinc from the water (about 35%). At day 21, the performance of all inoculated plants was significantly higher in comparison to the non-inoculated plants: the B1- and B2-inoculated plants removed more than 90% zinc, the consortium-inoculated 81% and B3-inoculated plants 79% while the non-inoculated plants reduced the zinc concentration in the water with only 64%. When the pots were supplemented with 400 mg L^-1^ Zn, the efficiency of the plants to decrease the Zn concentration in water was lower (**Figure 4B**). On day 14, the Zn concentrations in the waters of the non-inoculated and consortium-inoculated plants were significantly different, being, respectively, 277 mg L^-1^ (32% removal) and 172 mg L^-1^ (66% removal). This difference was even more pronounced at the second sampling point: 207 mg L^-1^ Zn in water samples of non-inoculated plants and 65 mg L^-1^ Zn in case of consortium-inoculated plants. This corresponded to 48% removal by non-inoculated plants and 84% removal by the plants inoculated with the consortium. The other treatments differed less from the non-inoculated plants.

Nickel was added at 20 mg L^-1^ in the LC treatments and the plants seemed efficient in removing it from water. Significant differences were not observed between the inoculated and non-inoculated plants; the recorded Ni reductions fluctuated between 73 and 85% for all treatments (**Figure 4C**). At the second sampling point, the nickel removal for untreated, B1-and B2-inoculated plants increased to, respectively, 91, 91, and 94% while the B3-inoculated plants decreased the Ni concentration with 78% Ni in total. In the HC treatments (40 mg L^-1^ Ni) the capacity of plants to remove Ni from the water was lower (**Figure 4D**). Consortium-inoculated plants showed the highest efficiency: 66 and 73% decreases were recorded after, respectively, 14 and 21 days. B1- and B2-inoculated plants followed while B3-inoculated and non-inoculated plants demonstrated the lowest ability to remove Ni from water.

It is important to mention that although Cd could be measured in the samples from day 0, it could not be detected any more in all water samples taken after 14 days of incubation, independently of the initial concentration.

### Effect of Contaminants on Biomass and Leaf Stress Enzymes

**FIGURE 5:**
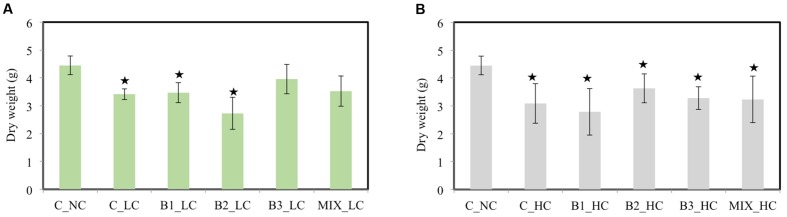
**The dry weight of the different treatments after 21 days when the low **(A)** and high **(B)** concentration of contaminants were added [data with asterisk are significantly different (*p* < 0.05) compared to the non-inoculated plants without any supplement of contaminants]**.

At the end of experiment, the plants were harvested and their dry weight was determined and compared among the treatments. When the LC of contaminants was added to the pots, the weight of the non-inoculated and the B1- and B2-inoculated plants was significantly affected compared to the weight of the unexposed control plants growing in absence of contaminants (**Figure 5A**). However, the dry weights of B3 and consortium-inoculated plants were not affected by the presence of the contamination mixture: the leaf biomass of those treatments were 2.8 and 2.7 g, respectively, and the leaf biomass of unexposed control plants was 2.9 g (data not shown). The weights of the inoculated plants were also compared with the weights of the non-inoculated plants under low level of exposure. Only the weight of the B2-inoculated plants was significantly lower in comparison with the non-inoculated plants while no significant differences were detected among the other treatments. A significant increase in root weight of B3-inoculated plants was observed in comparison to the weight of the non-inoculated ones (data not shown). Increasing the concentration of contaminants had a significant negative impact on the weight of all plants (**Figure 5B**) in comparison to the unexposed control plants while no significant differences were detected among the differences treatments of exposed plants. It is important to mention that the leaf weight of all plants was significantly affected compared to the unexposed control plants (data not shown). Only the root dry weight of non-inoculated plants (0.89 g) was significantly impacted by the presence of HCs of contaminants in comparison to the unexposed control plants (1.39 g).

**FIGURE 6:**
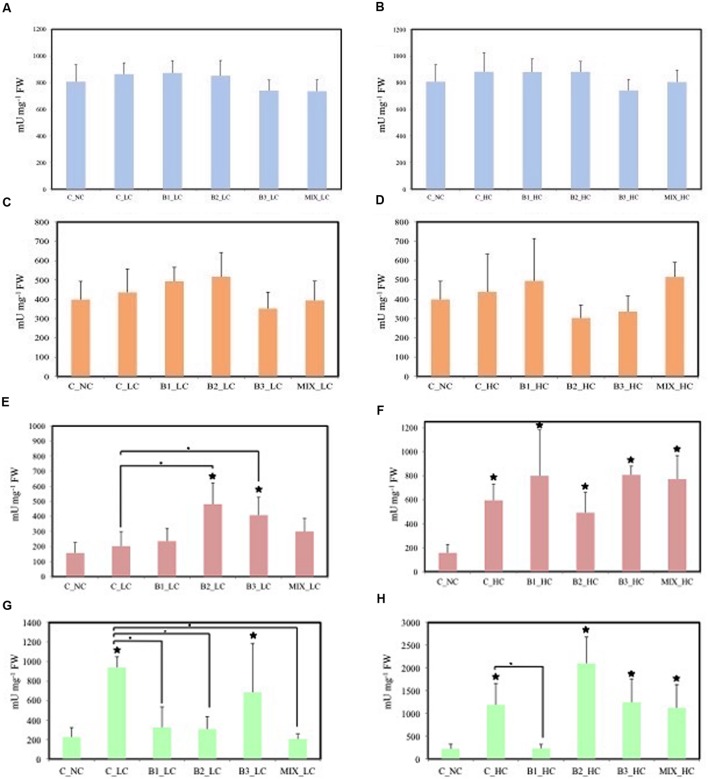
**Leaf stress enzymes of *J. acutus* of various treatments, **(A,B)** correspond to SOD, **(C,D)** correspond to GR, **(E,F)** correspond to GPOD, **(G,H)** correspond to SPOD [data with big asterisk are significantly different (*P* < 0.05) compared to the non-inoculated plants without any supplement of contaminants, small are significantly different (*p* < 0.05) compared to the non-inoculated plants with supplement of contaminants]**.

The effects of the different treatments on the activities of enzymes involved in anti-oxidative defense were determined in the leaves of the plants (**Figure 6**). In all treatments, the SOD and GR were not significantly affected by exposure to either LC or HC of contaminants. The activities of GPOD were significantly different between unexposed control plants and B2- and B3-inoculated plants at LCs of contaminants and between non-inoculated plants and the B2- and B3-inoculated plants. In HC treatments, the activity of this enzyme in leaves of all plants was significantly higher compared to the activity of the enzyme in leaves of unexposed plants. Significant increased activities of SPOD were recorded in leaves of non-inoculated and B3-inoculated helophytes in comparison to the unexposed control at LC of contaminants. Significant differences were also observed between the non-inoculated plants and B1-, B2-, and consortium-inoculated plants. The activity of these SPODs was significantly higher in leaves of non-inoculated, B2-, B3-, and consortium-inoculated plants in the HC treatments and only the activity in B1-inoculated plants was significantly lower than in the non-inoculated ones.

**FIGURE 7:**
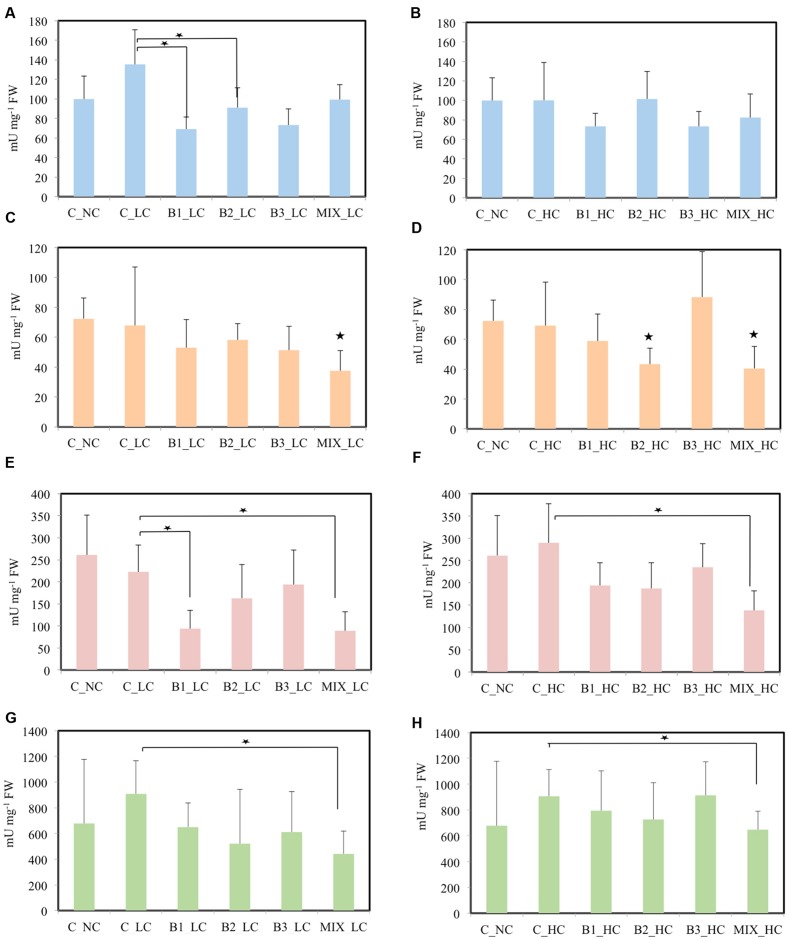
**Root stress enzymes of *J. acutus* of various treatments, **(A,B)** correspond to SOD, **(C,D)** correspond to GR, **(E,F)** correspond to GPOD, **(G,H)** correspond to SPOD [data with big asterisk are significantly different (*p* < 0.05) compared to the non-inoculated plants without any supplement of contaminants, small are significantly different (*p* < 0.05) compared to the non-inoculated plants with supplement of contaminants]**.

In the roots, significant differences in the activities of SOD were detected between the non-inoculated and B1- and B2-inoculated plants when the concentration of contaminants was low (**Figure 7**). At the higher contaminants concentration significant differences were not observed among the treatments. GR activity of plants inoculated with the consortium was significantly lower in comparison to unexposed plants in LC treatments. In HC treatments, the activity of GR was significantly affected in roots of B2- and MIX-inoculated plants in comparison with the unexposed control. The activities of GPOD and SPOD were significantly lower in the roots of consortium-inoculated plants compared to the non-inoculated plants at both levels of the contamination.

### Principal Component Analysis with the Root Stress Enzymes and Dry Weight toward the Contaminants

**FIGURE 8:**
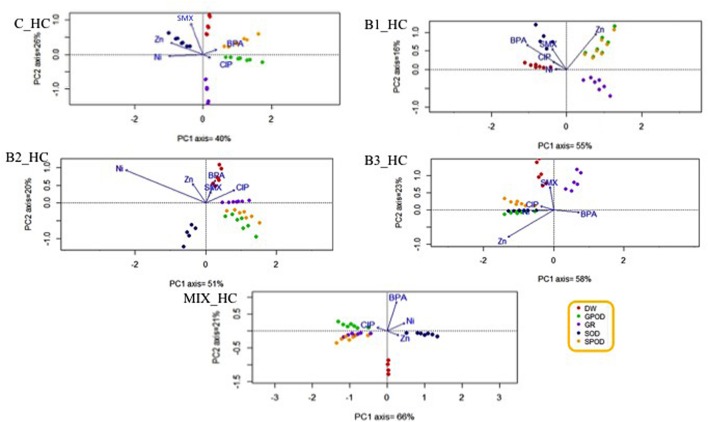
**Principal component analysis with the root stress enzymes, root dry weight, and the fitted environmental variables in high contamination treatments**.

**FIGURE 9:**
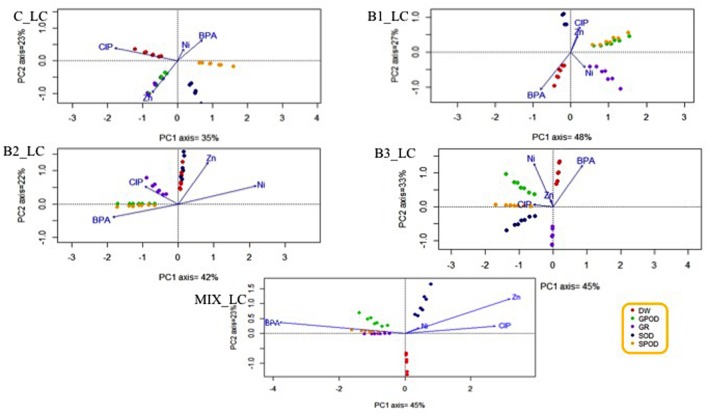
**Principal component analysis with the root stress enzymes, root dry weight, and the fitted environmental variables in low contamination treatments**.

Since the plant roots were in direct contact with all the contaminants applied in this experiments, the potential relationships between a contaminant and a specific root stress enzyme responses or root dry weight were investigated. The HC of organic contaminants and metals differently affected the various antioxidants (**Figure 8**), leading to the hypothesis that this effect may be treatment-specific. The same phenomenon was observed when the concentration of contaminants was low (**Figure 9**).

### Root Endophytic Bacterial Community and Diversity in Response to Contamination

**FIGURE 10:**
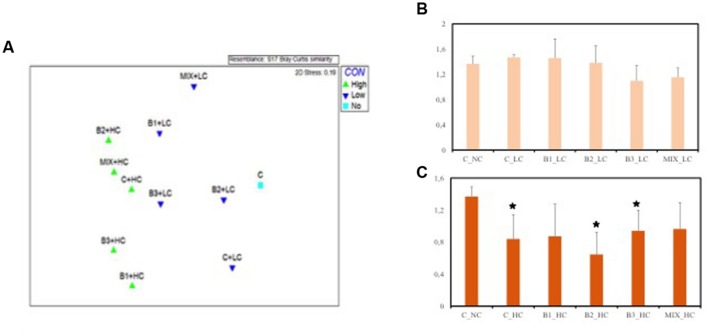
**(A)** Multidimensional scaling (MDS) ordination based on Bray–Curtis similarities from automated ribosomal intergenic spacer analysis (ARISA) fingerprints of *J. acutus* root communities under different levels of contaminants exposure. Shannon–Wiener diversity index among the different *J. acutus* root communities under low **(B)** and high **(C)** level of contaminants exposure [data with asterisk are significantly different (*p* < 0.05) compared to the non-inoculated plants without any supplement of contaminants].

ARISA fingerprints were analyzed in order to estimate the potential impact of the contamination and the inoculation effect on the bacterial diversities and community structures in roots at the end of the experiment. The MDS plot (**Figure 10A**) presents the distribution of the communities with the level of contamination as criterion and indicates that a separation occurred among the communities. The highest dissimilarity was observed between the bacterial communities of roots of plants growing in the highly contaminated pots and the roots of unexposed control plants (ANOSIM *R* = 0.334, *p* < 0.05). However, no significant differences were found among the root communities when the selecting factor was the inoculation effect. These results imply that the high level of contamination had a strong impact on the bacterial communities while the inoculated endophytes did not significantly alter the root communities.

Root bacterial diversity did not significantly differ among the treatments, although the diversity of the roots of non-inoculated, B1- and B2-inoculated plants seemed to be favored (not significantly) by the presence of the LC of contaminants in comparison to the endophytic community of unexposed plants (**Figure 10B**). When the plants were exposed to the HC of contaminants, decreases of the diversity were noticed for all root communities. However, only the non-inoculated, B2- and B3-inoculated roots harbored a significantly less diverse community compared to the unexposed control roots (**Figure 10C**).

## Discussion

Species of genus *Juncus* have been widely used in CWs treating various types of influents (Wu et al., 2014) such as industrial wastewater (Khan et al., 2009) and urban stormwater runoff (Ladislas et al., 2013). The species *J. acutus* is a common halophytic plant in Mediterranean ecosystems and has been studied for its high zinc tolerance and accumulation (Mateos-Naranjo et al., 2014; Santos et al., 2014). Moreover, this species has been found able to rhizofiltrate groundwater polluted with Cr(VI) and to accumulate it in the internal tissues (Dimitroula et al., 2015). Christofilopoulos et al. (2016) revealed the efficiency of *J. acutus* to treat contaminated water with organics (CIP, SMX, and BPA) and metals (zinc, nickel, chromium, and cadmium), especially when the concentrations of these contaminants were environmentally relevant.

In this study, the concentrations of the mixtures of contaminants that were used simulated hospital eﬄuents with high loadings and/or industrial eﬄuents. The ability of *J. acutus* to treat a variety of EOCs and metals was investigated. At such HCs (especially the HC treatments), it may become difficult for the plant to cope with the contaminants and its performance might decrease. As a possible solution for this, the potential increase of phytoremediation efficiency by inoculating endophytic bacteria with the appropriate traits was investigated. It has been demonstrated that inoculation of endophytic bacteria can positively influence the outcome of phytoremediation processes in areas contaminated with organics and/or metals (Weyens et al., 2010, 2013; Babu et al., 2015; Ma et al., 2015b). It is important to mention that the *J. acutus* plants in this experiment were grown on gravels and only irrigated with tap water in order to inhibit the growth of rhizospheric bacteria; due to this experimental set-up, the only carbon sources were the organic contaminants and the root exudates.

The effect of inoculation with rhizospheric or endophytic bacteria on plants growing on contaminated soils has been investigated by several studies (Rajkumar et al., 2009; Ma et al., 2011; Abhilash et al., 2012). In most of the these studies the beneficial effects of endophytic inoculants were highlighted, accomplished through promotion of plant growth and protection from stress factors (Becerra-Castro et al., 2013; Mesa et al., 2015) and even through enhancement of metal translocation in plant tissues (Babu et al., 2013; Ma et al., 2015a). With respect to organic xenobiotics, the role of endophytic bacteria in detoxification processes is of high importance since plants are photoautotrophic organisms lacking C metabolization pathways suitable for contaminant degradation, and only exhibiting contaminant transformation or immobilization mechanisms (Weyens et al., 2009b). For example, reductions of toluene or trichloroethylene (TCE) evapotranspiration were demonstrated after inoculation with a natural endophyte equipped with the pTOM toluene-degradation plasmid (Barac et al., 2004; Weyens et al., 2009a). Although CWs are systems that exploit the interactions between plants and their associated microorganisms in order to clean contaminated water, few studies have investigated the potential impact of endophytic bacteria inoculation in the performance of these systems (Shehzadi et al., 2014; Ijaz et al., 2015).

Mixtures of organic and inorganic contaminants at two different concentrations were selected for our experiment. In the LC treatments, the plant itself with the indigenous microbial community managed to cope with the contaminants and the differences in performances among the different inoculations depended on the type of the contaminant. For example, the capability of non-inoculated and inoculated plants to degrade BPA was similar while all inoculated plants demonstrated a better performance to remove antibiotics. As shown in a previous study (Syranidou et al., 2016), the indigenous community is highly enriched with BPA tolerant strains and potential degraders and this plant can efficiently clean BPA contaminated groundwater in short period of time and this phenomenon may have a positive impact on BPA removal from the water. Moreover, all the inoculated plants (especially B1- and B2-inoculated) enhanced zinc removal from water after 21 days compared to the non-inoculated even though *J. acutus* has been characterized as a potential zinc hyperaccumulator (Santos et al., 2014). Concerning nickel removal, the non-inoculated plants, B1- and B2-inoculated plant decreased nickel concentrations in water at a same extent while the nickel concentration in the B3 and consortium-inoculated pots was higher.

Adding nickel resistant root endophytic inoculants negatively influenced the root biomass and nickel accumulation in the Ni-hyperaccumulator *Noccaea caerulescens*; however, co-inoculation of specific strains had positive effects on plant biomass and Ni translocation to internal tissues (Visioli et al., 2015). Similarly, the dry weight of *J. acutus* inoculated with the consortium was not significantly different from that of the unexposed plants (**Figure 6A**). Moreover, the activity of the anti-oxidative enzymes in the roots of the consortium-inoculated plants was lower (in some cases significantly) compared to the non-inoculated plants. This indicates a protective role of the consortium against the oxidative stress induced by the different contaminants. As shown by other studies (Belimov et al., 2005; Qin et al., 2014; Thijs et al., 2014), the IAA and ACC deaminase production by the B1 and B3 strains along with other PGP traits may participate in alleviating the stress and improve plant growth at the same time.

When the contaminant concentration increased, the beneficial role of the inoculated consortium to the plants is more pronounced in terms of removing specific contaminants from the surrounding water and stress alleviation. The decrease of the concentrations of the organic xenobiotics in the water followed the same general pattern as in the LC treatments. The performance of the plants in decreasing the BPA concentration in water was similar while the inoculated plants showed enhanced antibiotics removal. SMX could not be detected in water samples of consortium-inoculated plants; it can be hypothesized that this greater SMX removal can be attributed to the endophytic consortium since the B3 strain has been characterized as a potential SMX degrader and B1 as a SMX tolerant strain. With respect to metals, the consortium-inoculated plants significantly decreased the zinc concentration in the water and slightly enhanced nickel removal in comparison to the non-inoculated plants. Moreover, cadmium was efficiently removed by all plant treatments. In several studies, species belonging to the genus *Juncus* have been found able to accumulate cadmium mainly in their belowground tissues (Ladislas et al., 2013; da Silva et al., 2015; Christofilopoulos et al., 2016).

The weight of all plants was significantly affected by the HC of xenobiotics and metals while the root dry weight of all inoculated plants did not differ significantly from unexposed plants. Moreover, the activities of all measured anti-oxidative enzymes in the roots of consortium-inoculated plants was (from slightly to significantly) lower compared to the activity in the roots of non-inoculated plants. These results indicate that the inoculated consortium may assist the plant to cope with elevated environmental stress and that this effect is more prominent in roots that are in direct contact with the contaminants in comparison to leaves. These findings are in accordance with other studies (Khan et al., 2015; Zhang et al., 2015; Li et al., 2016). Bioaugmentation with a selected consortium of two endophytic bacterial strains promoted the host plant growth and reduced the crude oil levels in soil (Fatima et al., 2015). In another study, bulk soil, rhizosphere and endophytic strains of *A. pseudoplatanus* were selected according to their plant growth potential and trinitrotoluene (TNT) transformation capabilities and formed a consortium (Thijs et al., 2014). When this consortium was inoculated to *A. capillaris*, it protected the grass from oxidative stress and contributed to TNT transformation. In this study, we noticed that the level of contamination together with the type of contaminant affect the physiological status of either inoculated or non-inoculated plants in a different way.

The inoculations that we performed did not alter the root endophytic community; it changed only in response to the different levels of contamination. This is in agreement with the observation that not the inoculation with *B. phytofirmans* but the additions of different metal immobilizers influenced the composition of shoot and rhizosphere communities (Touceda-González et al., 2015). Plant species and soil type seem to be crucial factors in shaping the endophytic communities (Phillips et al., 2008; Bulgarelli et al., 2012). Plants may have the ability to regulate the *in planta* bacterial catabolic genotypes in relation to the level of environmental contamination (Oliveira et al., 2014) while the entrance and transport of contaminants to various plant parts may also affect the endophytic microbiome (Su et al., 2015; Eevers et al., 2016). Instead of waiting for the contaminants to enter the plant and induce the development of corresponding contaminant biodegraders, we propose to proceed with bioaugmentation of the indigenous microbes that are carriers of appropriate genes to assist their host plant to better cope with the stress induced by the contamination and to earlier remediate the contaminated area/water.

With respect to diversity index, no statistical differences were detected among the root communities exposed to the low contamination (**Figure 10B**). At the highest level of contamination the microbial diversity of non-inoculated, B2- and B3-inoculated roots was negatively affected in comparison to the unexposed control roots. It seems that the roots inoculated with B1 strain and the consortium harbored a diverse microbiome that was less affected by the high levels of contamination.

## Conclusion

Inoculation with indigenous endophytic bacteria and especially the consortium was shown to have positive effects on the plant in terms of contaminant removal and stress alleviation. Also, the root communities were only altered in function of the contamination level. To our knowledge, this is the first study in which a positive impact on removal of EOCs together with metals was demonstrated after inoculation with endophytic bacteria. This study reveals that the exploitation of wetland helophytic plants and their associated bacteria shows promising potential toward implementing CW systems on a large scale with improved performance.

## Author Contributions

ES performed most of the experimental work. SC and GG did most of the chemical analysis work (measuring the organics and metals in water). ST has a profound experience in plant–microbe interactions and helped with discussion of the statistics. ES, ST, NW, JV, and NK contributed in the design of the experiments, ST, NW, JV, and NK helped with the proofreading of the manuscript. ES wrote the first draft manuscript.

## Conflict of Interest Statement

The authors declare that the research was conducted in the absence of any commercial or financial relationships that could be construed as a potential conflict of interest.
